# Workplace loneliness and the communication climate of healthcare workers: the moderating role of perceived social competence

**DOI:** 10.1186/s12913-025-13911-2

**Published:** 2025-12-18

**Authors:** Hüseyin Tolga Çağatay

**Affiliations:** Department of Medical Services and Techniques, Başkent University Vocational School of Health Services, Ankara, Türkiye

**Keywords:** Workplace loneliness, Emotional deprivation, Lack of social companionship, Organizational communication climate, Perceived social competence, Healthcare professionals, Moderation

## Abstract

**Background:**

Workplace loneliness has emerged as a significant challenge for healthcare systems, with consequences extending beyond employee wellbeing to organizational communication and patient safety. This study investigates how two dimensions of workplace loneliness –emotional deprivation (ED) and lack of social companionship (LSC)– relate to organizational communication climate (OCC), and whether perceived social competence (PSC) and demographic characteristics moderate these associations.

**Methods:**

Data were collected from 391 healthcare professionals working in two university hospitals in Türkiye using validated scales. Moderation analyses were conducted with Hayes’ PROCESS Macro (Models 1 and 2) and bootstrapping (5,000 resamples) to examine hypothesized effects.

**Results:**

Emotional deprivation was negatively associated with the organizational communication climate, b=-1.450, SE = 0.223, 95% CI [-1.888, -1.012], *p* < .001. Perceived social competence was positive, b = 0.739, SE = 0.189, 95% CI [0.368, 1.110], *p* < .001, and their interaction was significant, b = 0.070, SE = 0.027, 95% CI [0.016, 0.123], *p* = .012. Model R²=0.321, F(3,387) = 60.948, *p* < .001. In a parallel model, lack of social companionship was negatively associated with the communication climate, b=-2.761, SE = 0.155, 95% CI [-3.066, -2.456], *p* < .001, and perceived social competence was positive, b = 0.809, SE = 0.145, 95% CI [0.523, 1.094], *p* < .001, while their interaction was not significant, b=-0.003, SE = 0.023, 95% CI [-0.048, 0.043], *p* = .910. Model R²=0.543, F(3,387) = 153.476, *p* < .001. Demographic moderation analyses indicated small effects. For emotional deprivation, individual interactions with gender and marital status were not significant, ΔR²=0.001, *p* = .709, and ΔR²=0.010, *p* = .126, and the ED×PSC increment was modest, ΔR²=0.011, *p* = .083, the combined interaction block in the marital status model was significant, Both ΔR²=0.022, *p* = .030, with overall model R² between 0.331 and 0.344. For lack of social companionship, two demographic interactions reached significance with small variance gains, LSC×Gender ΔR²=0.002, *p* = .045, and LSC×Marital status ΔR²=0.003, *p* = .030, others were not significant, all ΔR²≤0.010 and *p* ≥ .057, with overall model R² between 0.556 and 0.565.

**Conclusion:**

Findings highlight that workplace loneliness – particularly LSC – is linked to unfavorable communication climates in healthcare settings. PSC functions as an individual resource that mitigates ED-related risks but is insufficient when structural companionship deficits exist. These results emphasize the need for dual-track interventions that build individual social capacities while fostering inclusive communication networks. Enhancing OCC may ultimately support staff wellbeing, institutional resilience, and patient safety.

**Supplementary Information:**

The online version contains supplementary material available at 10.1186/s12913-025-13911-2.

## Background

Healthcare relies on multidimensional communication among diverse employees. Although social interaction is inherent to care delivery, some healthcare workers experience workplace loneliness due to personal factors, job demands, unit constraints, and organizational structures [1–3]. This study is theory driven. Workplace loneliness is expected to erode trust, information exchange, and voice behavior, which are foundational elements of the organizational communication climate, OCC. OCC is therefore the proximal lens through which loneliness manifests in clinical teams, and it represents a unit level lever that managers can diagnose and change through everyday communication routines [4, 5]. Based on this logic, we posit that higher loneliness will relate to a less supportive OCC.

In healthcare, heavy workload, stress, and time pressure heighten loneliness and strain communication, which elevates safety risks and emotional costs for staff [4, 6–9]. Based on this evidence, the model predicts that higher workplace loneliness relates to a less supportive OCC. Focusing on OCC is consequential, because OCC captures openness, timeliness, accuracy, and psychological safety in information flow, which are directly linked to patient centered care and operational performance in hospitals [10, 11].

Workplace loneliness is modeled with two validated facets, emotional deprivation, ED, and lack of social companionship, LSC. ED is the felt absence of close, trusting bonds at work. LSC is the scarcity of everyday affiliative contact, for example missing a colleague to talk with during breaks or quick handoffs. This two factor structure has distinct nomological patterns, ED maps onto the quality of relational bonds, LSC maps onto the availability of weak ties and casual interaction [12]. In communication dependent clinical work, ED is expected to reduce warmth, trust, and speaking up. LSC is expected to thin informal channels that carry quick clarifications and coordination cues. Both processes predict a colder OCC. Recent reviews underline the practical value of the dual structure in contemporary workplaces, including healthcare [13, 14].

Perceived social competence, PSC, is introduced as a boundary condition. PSC reflects beliefs that one can initiate, sustain, and repair interpersonal exchanges and communicate effectively under demand. Social Cognitive Theory holds that people integrate observed interactions with efficacy beliefs, then adapt behavior to context, which supports clarity seeking and early help requests in time pressured teams [15, 16]. Employees with higher PSC are more likely to articulate needs, invite feedback, and participate in briefings and huddles, behaviors that preserve trust, information sharing, and voice under strain [17, 18]. The model therefore predicts that PSC buffers the negative links from ED and LSC to OCC.

OCC is not only theoretically central, it is also actionable. Short multidisciplinary huddles, escalation standards, and feedback loops are associated with improvements in information flow, psychological safety, and safety outcomes. This supports positioning OCC as the outcome of interest for unit level intervention design [19–21].

Demographic characteristics are treated as covariates to preserve parsimony and avoid abrupt model expansion. Gender, marital status, education, profession, and clinical unit shape opportunities for interaction and workload, and can shift perceived climate. They are included to adjust for background heterogeneity. Any demographic by loneliness probes are examined only as exploratory checks outside the core model (see Supplementary File S1).

### Purpose and hypotheses

This study examines how ED and LSC relate to OCC and tests whether PSC attenuates these links. The focus on OCC specifies a mechanism from psychosocial experience to unit level communication conditions that underpin coordination and patient care. The model aligns with recent evidence connecting stronger communication climates to measurable improvements in safety, retention, and service quality in hospitals [19, 21, 22]. Accordingly, the following hypotheses are advanced within the main text, with extended rationale provided in the literature review and hypothesis development (see Supplementary File S1).

#### H1

Loneliness at work due to emotional deprivation is negatively associated with organizational communication climate.

#### H2

Loneliness at work due to lack of social companionship is negatively associated with organizational communication climate.

#### H3

Perceived social competence moderates the association between emotional deprivation and the organizational communication climate, such that the negative association is weaker at higher levels of perceived social competence.

#### H4

Perceived social competence moderates the association between lack of social companionship and organizational communication climate.

Exploratory probes reported outside the core model.

#### H5

Demographic characteristics (gender, marital status, educational attainment, profession, and work unit), in conjunction with perceived social competence, may moderate the association between emotional deprivation and organizational communication climate.

#### H6

Demographic characteristics (gender, marital status, educational attainment, profession, and work unit), in conjunction with perceived social competence, may moderate the association between lack of social companionship and organizational communication climate.

**Fig. 1:**
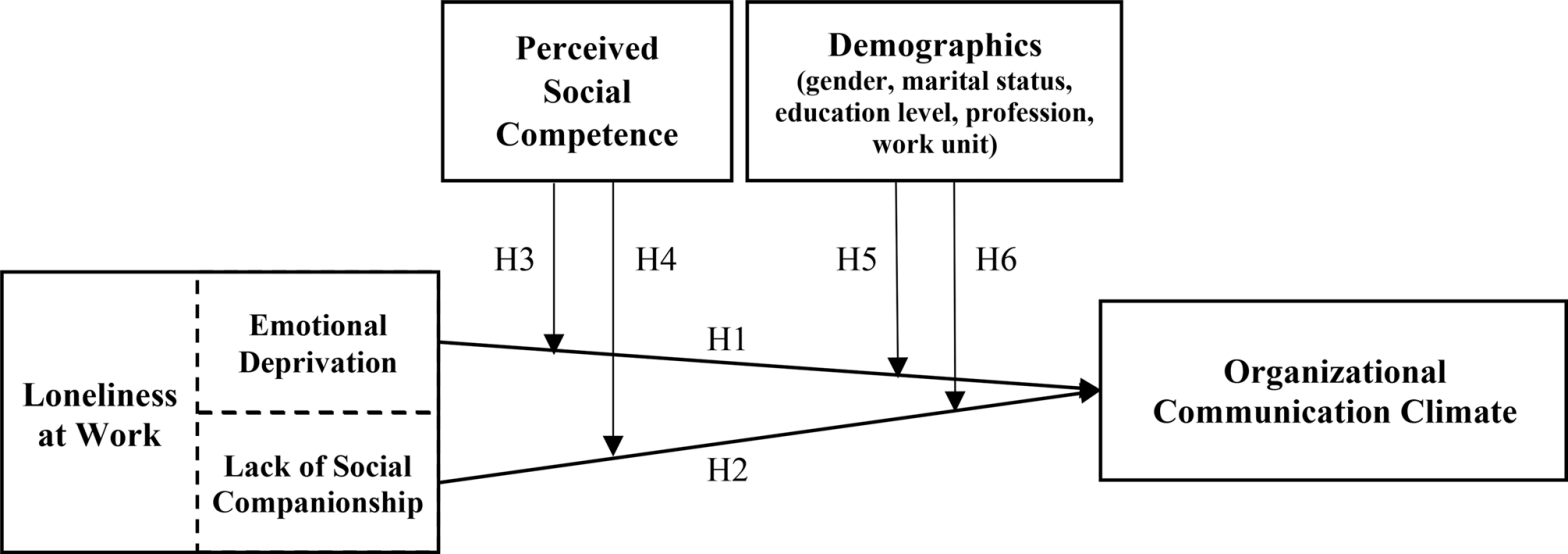
Basic model

These hypotheses aim to explore how the perception of social competence relates to feelings of loneliness and their associations with the communication climate in the workplace. The model illustrating these hypotheses is presented below, Fig. 1.

## Methods

### Participants

The population of this study consists of healthcare workers employed in two university hospitals located in Izmir province. Izmir is the third most populous city in Türkiye, which provides a significant metropolitan context for the study. According to the information obtained from the relevant units of the hospitals, there are a total of 983 people working in the human health hospital services class. Certain inclusion criteria were defined for the study: (a) Having at least one year of experience in the institution where they work, (b) Having a permanent position in the hospital, (c) Being included in the class “86.1-Hospital Services” under the code “86-Human Health Services” according to the NACE coding system, which is used as a reference in the production of statistics on economic activities in Europe, in order to be accepted as a health worker, (d) Providing informed consent to participate in the study. Exclusion criteria are: (a) Less than one year with the current hospital, (b) Working in a contract or temporary position at the hospital. According to the inclusion and exclusion criteria, the population was defined as 632 people. No sampling method was used in the study; the goal was to reach the entire population (*N* = 632).

At the end of the data collection process, valid data were obtained from 391 out of 632 healthcare professionals working in two hospitals in Izmir province. This corresponds to a participation rate of 62%, which is considered acceptable in organizational research and sufficient for generalizability [23]. It is believed that factors such as employees’ workload, the voluntary nature of the study, and occasional reluctance to participate individually played a role in this decrease in participation rate. Nevertheless, the final sample size adequately represents the defined population.

### Data collection procedure

The data were collected between January and February 2025 through an online survey administered via a secure institutional platform. Prior to distribution, the study protocol was reviewed and formally approved by the hospitals’ ethics committee. Hospital administrations appointed liaison officers to coordinate communication with employees. To maximize participation, invitation e-mails were sent to institutional addresses, posters were placed on staff noticeboards, and short announcements were made during departmental meetings. Each invitation contained a unique survey link, an explanation of the study’s purpose, and a statement on voluntary participation and confidentiality.

Before accessing the survey items, participants were required to read and electronically approve the informed consent form. The survey system was configured to prevent duplicate entries by restricting multiple submissions from the same IP address. Respondents could complete the questionnaire using personal or institutional devices (computers, tablets, smartphones) at times convenient to them, minimizing work disruption. To address non-response, reminder emails were sent twice at one-week intervals during the data collection window.

To ensure confidentiality, no identifying information (such as names, staff IDs, or IP logs) was stored. All responses were anonymized and stored on a secure server accessible only to the research team. Participants were explicitly informed that data would be analyzed in aggregate form and would not affect their employment status.

### Instruments

Demographic data were collected through a self-administered questionnaire. The questionnaire asked about gender, age, marital status, education level, profession, unit worked in, who do you live with, years in the profession, and years with current organization.

### Loneliness in work life scale

The scale developed by Wright, Burt, and Strongman [12] consists of a total of 16 items, including two sub-dimensions: “emotional deprivation” and “social companionship”. The scale was adapted into Turkish and its validity and reliability study was conducted by Doğan, Çetin, and Sungur [24]. The “Emotional Deprivation” sub-dimension of the scale consists of items 1–9 and the “Social Companionship” sub-dimension consists of items 10–16. An example item for the Emotional Deprivation dimension is: “I am satisfied with my relationships at work. An example item for the Social Companionship dimension is: “There is someone at work with whom I can discuss my daily work-related problems if necessary. The Loneliness at Work scale is a 5-point Likert-type scale ranging from “strongly disagree” to “strongly agree”. Some items on the scale are reverse coded; these reverse coded items are items 5, 6, 10, 11, 12, 14, 15, and 16. Scores that can be obtained from the scale range from 16 to 80, with high scores indicating high levels of loneliness at work and low scores indicating low levels of loneliness.

### Organizational communication climate scale

This scale, developed by Ballı and Ateş [25], consists of 30 items and is applied with a 5-point Likert-type rating system. It has two subdimensions: manager-based organizational communication (16 items: 1, 2, 4, 5, 7, 8, 11, 13, 17, 19, 20, 21, 22, 23, 28, 29) and employee-based organizational communication (14 items: 3, 6, 9, 10, 12, 14, 15, 16, 18, 24, 25, 26, 27, 30). For example, the statement “Management trusts the organization’s employees in business-related matters” represents the manager-based subdimension, while the statement “Employees in the organization are honest with each other about business” represents the employee-based subdimension. High scores on the scale indicate a positive and open communication climate, while low scores indicate a negative and closed communication climate.

In this study, the total score of the scale was used instead of its subdimensions in order to address the communication climate in a holistic manner. Conceptually, organizational communication climate reflects a shared, unit-level perception of how openly, accurately, and safely information flows within the organization, which aligns with climate theory that treats climates as collective appraisals of specific domains [10, 26, 27]. The decision to use the total score of the Organizational Communication Climate Scale was guided by both theoretical and practical considerations. First, organizational communication climate is conceptually understood as a shared, overarching perception that emerges from various interpersonal exchanges within the organization, including those with supervisors and peers. Treating the construct holistically allows us to capture this integrated perception rather than examining fragmented components. Second, focusing on the overall climate avoids unnecessary model complexity and maintains statistical power, which is particularly important when testing moderated effects with multiple predictors. Third, preliminary analyses showed that the two subdimensions were strongly correlated (*r* = .895, *p* < .001), supporting the validity of a higher-order factor structure that justifies the use of a composite score. Finally, from an applied perspective, healthcare organizations are typically more interested in the overall communication environment than in its specific facets when designing interventions and policies. For these reasons, the total scale score was used to examine the global dynamics of organizational communication climate in this study. The scale’s internal consistency coefficient was determined to be 0.96.

### Perceived social competence scale

The Perceived Social Competence Scale, which was developed by Anderson-Butcher, Iachini, and Amorose [28] and adapted to Turkish and analyzed for validity and reliability by Sarıçam, Akın, Akın, and Çardak [29], is a measurement tool that assesses the concept of social competence based on how individuals perceive themselves in social relationships and provide information about themselves. The scale consists of 6 items and has a 5-point Likert-type rating (ranging from “1 - Strongly Disagree” to “5 - Strongly Agree”). Sample scale item: “I get al.ong well with other people.” The range of scores that can be obtained from the scale is from 6 to 30, and there are no reverse-scored items. High scores on the scale indicate a high level of perceived social competence [29]. The internal consistency reliability coefficient of this scale was calculated to be 0.80.

### Data analysis

SPSS 26.0 and AMOS 23 software were used for statistical analyses. The construct validity of the scales was evaluated using confirmatory factor analysis. Fit indices were within recommended ranges, full results are provided in “Supplementary File S2” [30, 31]. Frequency analysis was used for descriptive statistics. Prior to conducting the main analyses, the assumptions of normality were tested. Skewness and kurtosis values for all continuous variables were examined and found to fall within the acceptable range of ± 1.5, as suggested in the literature [32]. Additionally, visual inspections of histograms and Q-Q plots supported the assumption of normal distribution. These results indicated that the data met the criteria for normality, justifying the use of parametric statistical analyses. Subsequently, the “Model 1 and 2” option of the PROCESS macro (version 4.0) in the SPSS program was used to test the hypotheses [33]. The bootstrapping method with a 95% confidence interval (based on 5000 bootstrap samples) was used to determine the significance of the conditional effects. The significance level for all analyses was set at *p* < .05.

### Multicollinearity control

To ensure the statistical validity of our model, it was important to assess whether the two sub-dimensions of workplace loneliness, emotional deprivation and lack of social companionship, exhibit problematic multicollinearity. These two constructs, although conceptually related, are theoretically distinct and capture different aspects of workplace loneliness. Emotional deprivation reflects the absence of deep, meaningful emotional connections at work, while lack of social companionship pertains to the lack of informal social interactions [12, 34].

To examine whether these dimensions could be treated as independent predictors in the model, multicollinearity diagnostics were conducted through linear regression analysis. The results indicated that the Variance Inflation Factor (VIF) values for both emotional deprivation and lack of social companionship were 1.063, well below the critical threshold of 5.0, and the Tolerance values were 0.941, which is well above the recommended minimum of 0.20 [35]. These results suggest that multicollinearity is not a concern for our model. Furthermore, the Pearson correlation coefficient between the two sub-dimensions was found to be *r* = .243 (*p* < .01), indicating a weak positive relationship between emotional deprivation and lack of social companionship. While these two dimensions are related, they remain empirically distinct, allowing for their separate inclusion as predictors in the model. Thus, based on both theoretical reasoning and statistical evidence, emotional deprivation and lack of social companionship were treated as independent predictors, with no significant multicollinearity issues present.

### Common method bias

In order to ascertain whether common method bias represented a potential issue in the study, we employed Harman’s single factor test, a widely utilised assessment tool in situations where self-report measures are involved. A factor analysis was conducted using SPSS to calculate the first eigenvalue from the data matrix. The presence of common method bias is indicated when a single factor or the first component accounts for the majority of the variance. The analysis revealed that the first eigenvalue explained 32.03% of the total variance, which is below the critical threshold of 50% for concern.

To strengthen this assessment, the study applied additional techniques recommended in recent methodological literature [36]. First, a marker variable, theoretically unrelated to the focal constructs, was included to test whether variance attributable to method bias would be absorbed. Estimates of the focal relationships remained stable after inclusion of the marker. Taken together, these checks indicate that common method bias is unlikely to threaten the validity of the findings.

## Results

The results of the socio-demographic characteristics of the study participants are shown in Table 1. The number of participants in the study was 391. 29.2% of the participants were male, 70.8% were female, and the mean age was calculated to be 31.3 years (SD = 7.638). Regarding marital status, 75.4% of the participants were married and 24.6% were single. In terms of educational level, the majority of participants were bachelor’s degree holders (54.7%), followed by associate’s degree holders (32.5%). While the rate of high school graduates is 2.6%, the rate of postgraduate graduates is 10.2%. The analysis of the living conditions of the participants showed that 74.9% lived with their families, 22.3% lived alone and 2.8% lived with friends. The average number of years in the profession was found to be 9.55 years (SD = 7.276), and the average number of years in the current institution was found to be 7.66 years (SD = 6.037).

**Table 1:** Socio-demographic characteristics of participants

Socio-demographics	*n*	%	Mean	SD
Gender				
Male	114	29.2		
Female	277	70.8		
Age	391		31.3	7.638
Marital Status				
Married	295	75.4		
Single	96	24.6		
Education Level				
High School	10	2.6		
Associate’s degree	127	32.5		
Bachelor’s degree	214	54.7		
Postgraduate degree	40	10.2		
Profession^*^				
Independent clinical decision makers (physician, dentist, pharmacist)	18	4.6		
Licensed allied care professionals (nurse, physiotherapist, dietician)	130	33.2		
Technical health personnel (technician, technologist)	243	62.1		
Unit worked in^**^				
Emergency Department	22	5.6		
Intensive Care Units	34	8.7		
Pediatrics Wards	16	4.1		
Internal Medicine Wards	43	11.0		
Surgical Wards	49	12.5		
Outpatient Clinics (Polyclinics)	62	15.9		
Support Services (Laboratory, Radiology, Pharmacy, etc.)	75	19.2		
Other Units (Administration, Counseling, etc.)	90	23.0		
Who do you live with?				
Alone	87	22.3		
With family	293	74.9		
With friends	11	2.8		
Years in the profession	391		9.55	7.276
Years with current organization	391		7.66	6.037

Note. * Profession was coded a priori using international classifications and scope of practice

Group 1 independent clinical decision makers (physicians, dentists, pharmacists)

Group 2 licensed allied care professionals (nurses, physiotherapists, dieticians)

Group 3 technical health personnel (technician, technologist)

This threefold scheme aligns with ISCO-08 major groups 22 Health Professionals and 32 Health Associate Professionals, the WHO Classifying Health Workers framework, and Eurostat methodology for health occupations [37–39]

Note.** “Unit worked in” was coded a priori by clinical acuity and workflow characteristics that shape communication. Emergency Department and Intensive Care Units, high acuity, time-critical care. Inpatient wards, Internal Medicine, Surgical, Pediatrics, distinct diagnostic and procedural flows. Outpatient Clinics, scheduled ambulatory care. Support Services, laboratory, radiology, pharmacy, and similar ancillary functions. This scheme is consistent with NHS descriptions of urgent and emergency care and ICU service standards, AHRQ settings of care, and OECD definitions of inpatient versus ambulatory services [40–42]

The analysis was conducted using Hayes’ PROCESS macro (Model 1) to examine the moderating role of perceived social competence in the associations between emotional deprivation, lack of social companionship, and organizational communication climate (Table 2). In the first model, emotional deprivation was found to be significantly and negatively associated with organizational communication climate (*B* = -1.450, *p* < .001), supporting Hypothesis 1. Perceived social competence showed a significant positive association with organizational communication climate (*B* = 0.739, *p* < .001). In addition, the interaction term (emotional deprivation × perceived social competence) was significant (*B* = 0.070, *p* = .012), indicating that perceived social competence moderates the association between emotional deprivation and organizational communication climate, thus providing support for Hypothesis 3. The model explains 32.1% of the variance in the dependent variable (R^2^ = 0.321).

In the second model, lack of social companionship was significantly and negatively associated with organizational communication climate (*B* = -2.761, *p* < .001), supporting Hypothesis 2. Similarly, perceived social competence was positively and significantly associated with organizational communication climate (*B* = 0.809, *p* < .001). However, the interaction term (lack of social companionship × perceived social competence) was not significant (*B* = -0.003, *p* = .910). This indicates that perceived social competence did not moderate the association between lack of social companionship and organizational communication climate, and therefore Hypothesis 4 was not supported. The second model explained 54.3% of the variance in the dependent variable (R² = 0.543).

**Table 2:** The moderating role of perceived social competence in the relationship between workplace loneliness and organizational communication climate

	Effect	SE	t	*p*	LLCI	ULCI
*Model 1*						
Constant	104.823	1.158	90.546	0.000	102.547	107.100
Emotional Deprivation	-1.450	0.223	-6.508	0.000	-1.888	-1.012
Perceived Social Competence	0.739	0.189	3.912	0.000	0.368	1.110
ED × PSC	0.070	0.027	2.539	0.012	0.016	0.123
R^2^ = 0.321; F (3, 387) = 60.948; *p* < .000						
*Model 2*						
Constant	103.579	0.897	115.498	0.000	101.816	105.342
Lack of Social Companionship	-2.761	0.155	-17.814	0.000	-3.066	-2.456
Perceived Social Competence	0.809	0.145	5.566	0.000	0.523	1.094
LSC × PSC	-0.003	0.023	-0.113	0.910	-0.048	0.043

Abbreviations. SE, Standard Error; t, t-value; p, significance; LLCI, Lower Level Confidence Interval; ULCI, Upper Level Confidence Interval

The results suggest that perceived social competence has a moderating role in the association between emotional deprivation and organizational communication climate, but this moderating effect does not emerge in the association between lack of social companionship and organizational communication climate. These findings highlight the differential role of perceived social competence on the effects of social deficits.

**Fig. 2:**
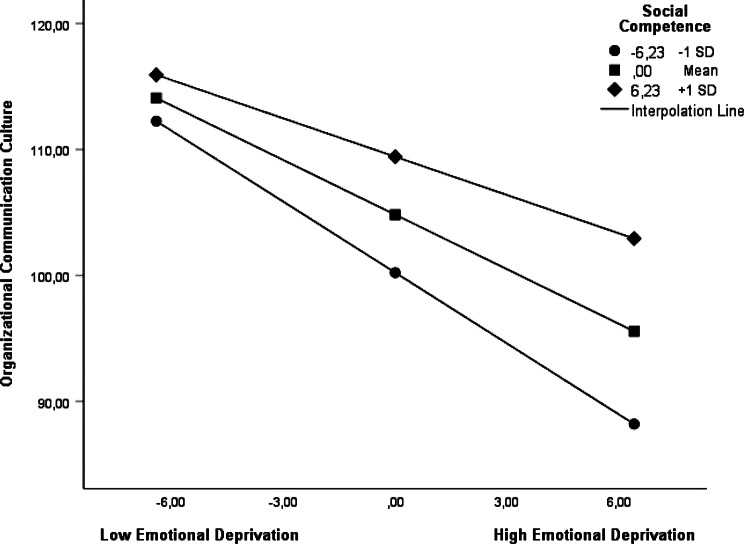
Interaction emotional deprivation and social competence as predictors on organizational communication climate

Figure 2 shows the interaction of emotional deprivation and perceived social competence on organizational communication climate. The results show that organizational communication climate is lower as emotional deprivation is higher. However, this association varies depending on the individual’s level of perceived social competence.

For individuals with high social competence, the negative association between emotional deprivation and organizational communication climate remains at a more limited level. These individuals are able to maintain communication climate to a great extent despite experiencing emotional deprivation. For individuals with moderate social competence, a more notable decrease in communication climate is observed as emotional deprivation increases. For individuals with low social competence, this negative association is most pronounced, and the relationship between emotional deprivation and organizational communication climate shows a stronger negative trend.

These results indicate that perceived social competence is an important moderator variable that significantly moderates the association between emotional deprivation and organizational communication climate. Individuals with high social competence appear more resilient in the face of emotional deprivation and are able to limit its negative associations with communication climate. On the other hand, individuals with low social competence are more vulnerable in this context, and their experiences of emotional deprivation are more strongly associated with a less favorable organizational communication climate.

The extended moderation analyses incorporating demographic variables (gender, marital status, education level, profession, and unit worked in) together with perceived social competence (PSC) were conducted to test Hypotheses 5 and 6 (Table 3).

For H5, which examined whether demographic characteristics, in conjunction with perceived social competence, are associated with variations in the link between emotional deprivation and organizational communication climate, the findings provided only limited evidence. emotional deprivation showed a consistent negative association with organizational communication climate across models (e.g., b=-1.96, SE = 0.67, *p* = .004), yet most interaction terms with demographic variables were not significant. The marital status model was the only case in which the combined interaction block (ED × PSC × Marital Status) accounted for a small but statistically significant increment in variance (ΔR²=0.022, *p* = .030). This pattern indicates that marital status, together with perceived social competence, may be related to differences in how emotional deprivation corresponds with organizational communication climate, while gender, education level, profession, and unit worked in did not demonstrate significant associations at the interaction level.

For H6, which tested whether demographic characteristics and perceived social competence jointly moderate the association between lack of social companionship and organizational communication climate, more consistent evidence was observed. lack of social companionship demonstrated a strong and robust negative association with organizational communication climate across all models (e.g., b=-3.09, SE = 0.17, *p* < .001). Regarding moderation, two demographic variables displayed significant interaction blocks: gender (ΔR²=0.002, *p* = .045) and marital status (ΔR²=0.003, *p* = .030). Education level also showed a trend-level effect (ΔR²=0.010, *p* = .057), whereas profession and unit worked in did not yield significant interaction effects.

**Table 3:** Moderating roles of perceived social competence and demographics in the associations between workplace loneliness and organizational communication climate

Model	*R*² / F(df1,df2)	Predictor	β (SE)	t	*p*	95% CI	ΔR² (Interaction Block)
ED + PSC + Gender	0.331 / 40.12 (10,380)*	ED	-1.36 (0.47)	-2.90	0.004	[-2.28, -0.44]	ED×PSC: 0.011 (*p* = .083) ns; ED×Gender: 0.001 (*p* = .709) ns
		PSC	0.73 (0.26)	2.76	0.006	[0.21, 1.25]	
ED + PSC + Marital Status	0.341 / 51.95 (10,380)*	ED	-2.88 (0.94)	-3.06	0.002	[-4.73, -1.03]	ED×PSC: 0.010 (*p* = .081) ns; ED×Marital: 0.010 (*p* = .126) ns; Both: 0.022 (*p* = .030)
		PSC	0.75 (0.26)	2.90	0.004	[0.24, 1.26]	
ED + PSC + Education Level	0.336 / 39.37 (14,376)*	ED	-1.96 (0.67)	-2.90	0.004	[-3.28, -0.63]	ED×PSC: 0.011 (*p* = .086) ns; ED×Education: 0.004 (*p* = .38) ns
		PSC	0.73 (0.26)	2.77	0.006	[0.21, 1.25]	
ED + PSC + Profession	0.332 / 34.23 (12,378)*	ED	-1.55 (0.81)	-1.92	0.056	[-3.15, 0.04]	ED×PSC: 0.011 (*p* = .080) ns; ED×Profession: 0.001 (*p* = .748) ns
		PSC	0.74 (0.27)	2.75	0.006	[0.21, 1.27]	
ED + PSC + Work Unit	0.344 / 20.94 (22,368)*	ED	-0.96 (1.09)	-0.88	0.378	[-3.11, 1.18]	ED×PSC: 0.009 (*p* = .129) ns; ED×Unit: 0.011 (*p* = .965) ns
		PSC	0.77 (0.27)	2.89	0.004	[0.25, 1.29]	
LSC + PSC + Gender	0.556 / 106.96 (10,380)*	LSC	-3.09 (0.17)	-18.23	< 0.001	[-3.43, -2.76]	LSC×PSC: 0.000 (*p* = .891) ns; LSC×Gender: 0.002 (*p* = .045)
		PSC	0.77 (0.22)	3.49	0.001	[0.34, 1.20]	
LSC + PSC + Marital Status	0.556 / 115.09 (10,380)*	LSC	-2.19 (0.34)	-6.35	< 0.001	[-2.87, -1.51]	LSC×PSC: 0.001 (*p* = .832) ns; LSC×Marital: 0.003 (*p* = .030)
		PSC	0.77 (0.22)	3.49	0.001	[0.34, 1.20]	
LSC + PSC + Education Level	0.565 / 95.04 (14,376)*	LSC	-1.43 (4.71)	-0.30	0.762	[-10.69, 7.83]	LSC×PSC: 0.001 (*p* = .814) ns; LSC×Education: 0.010 (*p* = .057)
		PSC	0.74 (0.22)	3.41	0.001	[0.31, 1.17]	
LSC + PSC + Profession	0.557 / 88.23 (12,378)*	LSC	-3.08 (0.67)	-4.57	< 0.001	[-4.40, -1.75]	LSC×PSC: 0.000 (*p* = .97) ns; LSC×Profession: 0.004 (*p* = .12) ns
		PSC	0.79 (0.22)	3.54	0.001	[0.35, 1.23]	
LSC + PSC + Work Unit	0.565 / 54.32 (22,368)*	LSC	-2.88 (0.27)	-10.48	< 0.001	[-3.41, -2.34]	LSC×PSC: 0.001 (*p* = .565) ns; LSC×Unit: 0.004 (*p* = .758) ns
		PSC	0.77 (0.22)	3.45	0.001	[0.33, 1.21]	

Overall, these results offer limited support for Hypothesis 5 and partial support for Hypothesis 6. perceived social competence consistently appeared as a positive predictor of organizational communication climate, while demographic variables showed modest and selective roles. Thus, workplace loneliness ‒whether conceptualized as emotional deprivation or lack of social companionship‒ appears to be linked to organizational communication climate in ways that may vary slightly depending on certain demographic characteristics, although these effects are generally small and context-specific.

## Discussion

This study examined the relationship between employees’ perceptions of the organizational communication climate, OCC, in healthcare settings and two dimensions of workplace loneliness, emotional deprivation, ED, and lack of social companionship, LSC. The study also examined whether perceived social competence, PSC, and selected demographic characteristics influenced these relationships. In all models, ED and LSC were negatively related to OCC, with LSC showing a stronger connection. These findings are consistent with the interpretation that OCC reflects shared patterns, norms, and ties in clinical teams [43, 44]. Deficits in day to day companionship, for example fewer informal interactions and sparser networks, are therefore more likely to register in OCC than purely affective deprivation [45, 46]. This view is also coherent with the dual structure of workplace loneliness, which distinguishes emotional deprivation from a lack of social companionship and links these facets to different communication channels at work [47].

The most important strength of the results is that they distinguish between ED and LSC. Although both dimensions were negatively correlated with OCC, the LSC–OCC association was stronger and more consistent across different models. This is consistent with the idea that, by design, OCC is a property of shared communication patterns, norms and ties. Thus, deficits in companionship (e.g. fewer informal interactions and sparser networks) may map more directly onto perceptions of climate than purely affective deprivation. This interpretation is consistent with healthcare dynamics where cross-disciplinary coordination is critical. Recent organisational and communication literature supports this logic, demonstrating that climates of open, supportive exchange strengthen identification and coordination, whereas relational frictions erode collective sensemaking and performance [11]. In healthcare, where cross-disciplinary handoffs, coordination under time pressure and team trust are vital, missing social ties plausibly reverberate through both communication quality and climate. Furthermore, healthcare research continues to demonstrate that social support mitigates strain and burnout ‒related constructs associated with climate‒ suggesting that deficits in companionship can exacerbate communication difficulties [48].

The moderating role of PSC clarifies these patterns further. While PSC consistently buffered the adverse influence of ED on OCC, its role in the LSC–OCC link was negligible, underscoring structural rather than individual pathways. One possible interpretation is that PSC, an individual resource associated with social efficacy, emotion regulation, and adaptive interaction strategies, helps employees reframe or compensate for unmet emotional needs. This allows them to sustain constructive communication behaviors with colleagues and supervisors. By contrast, when the core issue is a scarcity of LSC – an inherently structural or network problem – individual skill may be insufficient in the absence of relational density and supportive routines. Contemporary overviews of work loneliness emphasize this multilevel logic: personal resources matter, but network access and situational affordances are decisive for embeddedness [14, 49].

Beyond examining the direct associations among ED, LSC, OCC, and PSC, it is critical to consider OCC’s potential function as a mediating mechanism in linking loneliness to broader organizational outcomes such as job performance, work engagement, and organizational commitment. Recent meta-analytic evidence underscores the significant negative effects of work loneliness on these outcomes [14]. For instance, research indicates that social companionship mediates the relationship between organizational support and job performance, while workplace loneliness has been shown to undermine employee engagement and commitment, particularly when coworker exchange is weak [50, 51]. Additionally, studies reveal that isolation erodes communication quality, which in turn reduces trust – a core facet of OCC [52]. Integrating these insights emphasizes that a weakened communication climate is not merely an outcome of loneliness but may serve as a mechanism driving adverse organizational behaviors and experiences.

To investigate whether ED/LSC–OCC connections differ according to social positions frequently discussed in loneliness literature, a model was designed that incorporated demographic variables (gender, marital status, education level, profession, and work unit) alongside PSC. The results were selective. For ED, interaction blocks involving demographic variables were largely insignificant, with only a small joint effect emerging for marital status (ΔR² ≈ 0.022). This indicates limited heterogeneity when PSC is considered. For LSC, two interaction blocks (gender and marital status) reached significant levels, and education showed a trend-level effect. These modest changes are consistent with new evidence suggesting that the distribution and impact of loneliness may vary depending on gender and marital status. However, these findings vary across settings and life stages [53–55]. Occupational and unit effects were negligible in our data, consistent with recent cross-contextual studies emphasizing the importance of work conditions and broader social environments over occupational labels themselves [56, 57].

Taken together, these findings support a multilevel account of workplace loneliness and communication climate. At the individual level, personal competencies (e.g., PSC) are related to better communication experiences, especially when addressing emotional unmet needs. At the relational/structural level, the density and quality of social ties (the absence of LSC) seem to correspond more directly to OCC. Recent syntheses echo this multilevel view, stating that loneliness in organizational contexts is not merely an affective state, but rather a dynamic phenomenon situated within team structures, digital communication arrangements, and job design [14, 49]. In healthcare organizations, such sparse networks or restrictive norms can be especially detrimental, given the reliance on fast, reliable communication for patient safety. From a systems perspective, small individual differences rarely offset sparse networks or norms that limit casual interaction.

### Practical implications

The demographic moderation results suggest tailoring interventions to specific groups rather than segmenting the population broadly. Although female employees may report stronger links between loneliness and negative work attitudes in certain cultural or organizational contexts and unmarried employees may have fewer emotional support resources outside of work, the mostly null or minimal moderation effects observed and the robust direct effects of LSC indicate that interventions should be universally available yet flexibly delivered (e.g., peer support channels accessible across units and shifts) with ongoing monitoring to ensure equitable access regardless of gender or marital status. This is consistent with evidence indicating that family status influences loneliness, though its effects often interact with broader social and relational factors (e.g., recent findings on widowhood and loneliness trajectories) [58]. Furthermore, contemporary demographic research cautions against attributing loneliness solely to education, revealing that transitions and socioeconomic contexts play a more significant explanatory role [59, 60].

First, interdisciplinary team-building initiatives should be prioritized. Structured “huddles” at the beginning and end of shifts, interdisciplinary case reviews, and cross-unit problem-solving workshops can strengthen relational ties, enhance clarity, and reduce the isolation that often arises in fragmented care processes. Such practices align with evidence showing that regular, structured opportunities for peer exchange improve team identification and communication quality in healthcare [61]. Similarly, initiatives that increase interaction opportunities, such as peer mentoring and protected debriefs at shift changes, are likely to improve the climate where LSC is important. Research on communication climate shows that establishing predictable, psychologically safe channels for exchange strengthens identification and team coordination [11]. Specifically in healthcare, bolstering social support has been shown to lead to lower burnout and improved staff wellbeing, indicating a healthier communication environment [48].

Second, mentorship programs tailored to junior staff, residents, or newly hired nurses may buffer the effects of loneliness by embedding them into established communication networks. By pairing less experienced professionals with senior staff, healthcare organizations can provide both emotional support and role-specific guidance, reducing the risk of emotional deprivation. This is particularly relevant in high-stress units such as emergency and intensive care, where the combination of workload and emotional demand heightens vulnerability [62, 63].

Third, communication training customized to healthcare contexts should be developed. For example, simulation-based training for emergency departments can incorporate scripts for high-pressure interactions, while programs for medical secretaries may focus on managing hierarchical communication and patient-facing stressors. Tailoring content to unit-specific stressors ensures that employees acquire skills that are directly transferable to their daily routines. Such targeted training resonates with frameworks like the TeamSTEPPS program, which emphasizes communication, leadership, and mutual support as core competencies for patient safety [64].

Beyond these unit-specific initiatives, developing social and emotional competencies more broadly (e.g., perspective-taking, navigating conflict, and assertively and empathetically speaking up) may mitigate the association between ED and OCC. Micro-interventions that provide clinicians with language and scripts for difficult conversations can be incorporated into simulation-based training. Additionally, organization-wide efforts that recognize loneliness as both a public health and productivity concern can shift norms. Recent national advisories recommend strengthening social infrastructure in workplaces through inclusive meeting practices, mentorship, and community building [22, 65].

Finally, findings from this study can be linked to broader health workforce resilience frameworks. For instance, the World Health Organization’s framework highlights both individual-level capacity building and system-level reforms to improve working conditions [66]. Embedding loneliness reduction strategies within these frameworks could strengthen their impact and ensure alignment with institutional priorities such as patient safety, staff retention, and service quality.

For future research, adopting network-aware and multilevel designs would be highly informative. Sociometric mapping of advice and friendship ties, combined with repeated OCC measurements, could reveal how shifts in network structure relate to evolving communication climates. Multilevel designs could also determine whether interventions like structured huddles buffer the loneliness–communication link at individual and unit levels. Evaluating whether PSC training is most effective when coupled with structural – supports such as cross-disciplinary reviews or social connection initiatives – would address whether skills and opportunities operate complementarily [67, 68].

In summary, ED, and particularly LSC, were consistently linked with less favorable organizational communication climates among healthcare workers in our sample. PSC appeared to mitigate ED–OCC associations but had limited impact on LSC–OCC links. Demographic moderation was modest and selective, primarily involving gender and marital status. These findings support a dual-track intervention model: (1) institutional efforts to strengthen everyday social networks and normalize supportive communication practices, and (2) individual-level capacity building to help staff navigate emotionally laden situations. Combining both approaches promises to foster climates where information flows effectively, psychological safety is maintained, and cross-disciplinary collaboration thrives [47, 69].

### Limitations

Although the findings of this study provide meaningful insights into the associations between workplace loneliness and organizational communication climate, several limitations warrant careful consideration. First, the cross-sectional design restricts causal inference. While emotional deprivation and lack of social companionship were associated with less favorable communication climates, reverse pathways are equally plausible – for instance, a deficient or closed communication climate may foster employees’ feelings of isolation. Unmeasured common causes, such as leadership style or organizational culture, could also explain both loneliness and communication outcomes. As recent methodological reviews emphasize, future research should employ longitudinal, experimental, or mixed-method approaches to disentangle temporal ordering and underlying mechanisms, thereby strengthening causal interpretations.

Second, although demographic moderators (gender, marital status, education level, profession, and work unit) were systematically examined, most interaction effects were weak or non-significant. This pattern reduces concerns about strong confounding by demographic factors once perceived social competence and core loneliness dimensions were included. However, the possibility of subgroup-specific suppressor effects remains, and future research using larger, stratified samples may help clarify whether nuanced differences exist across demographic strata.

Third, all variables were measured through self-report questionnaires, which may introduce response biases such as social desirability and common method variance. While statistical checks indicated no problematic multicollinearity, reliance on subjective reporting may limit the precision of estimates, particularly in socially sensitive domains such as loneliness. Future research could integrate objective or behavioral measures, such as sociometric network analyses, communication audits, or digital trace data, to triangulate findings.

Another limitation concerns the scope of outcome variables included in the study. While our primary focus was to investigate the role of workplace loneliness and perceived social competence in shaping organizational communication climate, we did not examine broader organizational outcomes such as job performance, engagement, or job satisfaction. These constructs are theoretically relevant and could provide additional insights into the consequences of workplace loneliness if included in future research. Furthermore, the potential mediating role of OCC in linking loneliness to such outcomes should be empirically tested to strengthen the explanatory power of this line of research.

Fourth, the study context was geographically limited to two university hospitals in İzmir, Türkiye’s third-largest city. Although this setting provides a valuable lens on healthcare professionals, the generalizability of findings to other sectors, cultural contexts, or organizational forms remains uncertain. Loneliness and social competence are socially embedded phenomena, and their meanings may vary across societies, organizational structures, and job designs. Cross-cultural comparative research and replication across industries would therefore enhance the robustness of conclusions.

Finally, measurement decisions impose additional constraints. The Organizational Communication Climate Scale was operationalized as a composite score in this study, as extremely high correlations between its subdimensions indicated substantial overlap. While this choice was both conceptually and statistically defensible, it limited the ability to differentiate between managerial–employee versus peer communication processes. Future studies should examine these dimensions separately to illuminate whether workplace loneliness differentially affects top-down communication channels versus peer-level exchanges.

## Conclusion

This study demonstrates that workplace loneliness – conceptualized as emotional deprivation and lack of social companionship – is consistently linked to less favorable organizational communication climates among healthcare professionals. Both dimensions exerted negative associations, though lack of social companionship emerged as the stronger and more stable predictor. Perceived social competence buffered the emotional deprivation–climate link, while demographic variables had only modest and selective moderating effects, primarily for gender and marital status.

Theoretically, these findings advance the understanding of workplace loneliness by distinguishing between emotional and relational dimensions and clarifying their differential pathways into organizational communication climate. They highlight that while personal competencies such as social competence can mitigate emotional deprivation, relational deficits such as companionship loss are more directly tied to communication structures and norms.

Practically, the results emphasize a dual-track approach. At the institutional level, strategies that strengthen everyday social ties ‒such as structured interdisciplinary huddles, peer mentoring, and inclusive debriefing practices – can enhance communication climates. At the individual level, building social and emotional competencies may help employees manage emotionally demanding interactions. By addressing both structural and personal aspects of workplace loneliness, organizations can sustain climates that foster open information exchange, psychological safety, and effective collaboration.

In sum, the study underscores that workplace loneliness is not only an individual challenge but also a systemic issue. Addressing it requires simultaneous investment in organizational practices and individual capacities to support resilient and communicative healthcare environments.

## Supplementary Information

Below is the link to the electronic supplementary material.

Supplementary Material 1

Supplementary Material 2

## Data Availability

The data supporting the findings of this study are available from the corresponding author on reasonable request.

## Abbreviations

ED: Emotional Deprivation
LSC: Lack of Social Companionship
OCC: Organizational Communication Climate
PSC: Perceived Social Competence
CFA: Confirmatory Factor Analysis
VIF: Variance Inflation Factor
LLCI: Lower Level Confidence Interval
ULCI: Upper Level Confidence Interval
SE: Standard Error
SD: Standard Deviation
df: Degrees of Freedom
GFI: Goodness of Fit Index
AGFI: Adjusted Goodness of Fit Index
CFI: Comparative Fit Index
NFI: Normed Fit Index
TLI: Tucker–Lewis Index
SRMR: Standardized Root Mean Square Residual
RMSEA: Root Mean Square Error of Approximation
