# Supplementary material for: Workplace loneliness and the communication climate of healthcare workers: the moderating role of perceived social competence
Source: BMC Health Serv Res. 2025 Dec 18;26:106. doi: 10.1186/s12913-025-13911-2 (PMC12829023; doi:10.1186/s12913-025-13911-2)
Supplement: Supplementary file 1 — Supplementary Material 1 [file 12913_2025_13911_MOESM1_ESM.docx]

**SUPPLEMENTARY FILE-S1**

**Literature review and hypothesis development**

**Workplace loneliness, scope and dual structure**

Workplace loneliness is multifaceted, and two dimensions are central in contemporary research, emotional deprivation, the felt absence of close, trusting bonds at work, and lack of social companionship, the scarcity of day-to-day affiliative contact. Recent evidence synthesizing workplace samples shows that loneliness relates to poorer wellbeing and occupational functioning, and importantly, to modifiable features of work design and interaction routines, which makes the construct actionable in unit contexts [1]. Management scholarship likewise consolidates decades of findings and situates workplace loneliness within relational and structural mechanisms that play out inside teams and across units, strengthening the case for a dual structure that captures both deep relational deficits and everyday affiliative scarcity [2].

The concept of loneliness has long been defined as a subjective sense of relational deficit, which anchors the modern dual structure theoretically [3]. Scholars distinguish between chronic loneliness, persistent and trait like, and situational loneliness, context driven and temporary, a distinction that helps explain variability in workplace experiences over time [4]. Although situational loneliness may be less harmful in the long run, in both cases employees with weaker social communication skills are more likely to experience depressive moods that can intensify loneliness and impede workplace exchange [5]. In organizational settings, this experience manifests primarily in two facets, emotional deprivation and lack of social companionship. ED arises when the need for deep, meaningful relationships goes unmet. LSC reflects unmet needs for acceptance and everyday affiliative contact. In healthcare, fast-paced and high strain environments can heighten both patterns, as shown in recent occupational samples that link workload, strain, and loneliness in clinical teams [6].

These two facets carry different explanatory roles for communication dependent work. ED aligns with the quality of close bonds, predicts lower trust, and suppresses speaking up, which undermines the openness and psychological safety that characterize a supportive communication climate. LSC aligns with the availability of casual interaction and weak tie contact, predicts thinner informal channels, and reduces timely clarification and coordination cues across shifts and roles. Organizational behavior research shows that loneliness erodes affiliation and, through that route, reduces performance and collaborative exchange, which makes the dual structure theoretically meaningful for team communication processes [7]. Contemporary, futures oriented reviews reach a similar conclusion and recommend modeling ED and LSC separately when the outcomes depend on day-to-day information flow and team climate in complex services like healthcare [8].

**Organizational communication climate in healthcare**

Organizational communication climate, OCC, captures shared perceptions about how openly, accurately, and safely information moves across a unit. OCC is distinct from general organizational climate because it focuses specifically on information flow, voice, and psychological safety in communication. In hospitals, OCC shapes speaking up, error reporting, handoffs, and day-to-day coordination. Strong climates show timely exchange, clarity, and psychological safety. Weak climates show guarded talk, delayed escalation, and fragmented teamwork. Large-scale evidence links OCC to patient centered care implementation and operational performance, which establishes OCC as a consequential outcome in clinical units [9, 10].

Antecedents of OCC are relational and procedural. Leadership that models transparency and invites voice strengthens climate norms. Peer support and trust reinforce willingness to share concerns. Structured routines, for example briefings, check backs, and tiered safety huddles, convert these norms into predictable practice on busy wards. Reviews and multi-site studies report that teams with clearer communication norms coordinate faster and make fewer preventable errors, while poor climates exacerbate isolation and information loss during shift changes and crises [11–13].

Intervention evidence shows that OCC is actionable at the unit level. Practical guidance defines safety huddles as short, multidisciplinary briefings at a fixed time and place, focused on patients at risk and on daily hazards. Hospitals that adopt tiered huddles, escalation standards, and feedback loops report improvements in information flow, psychological safety, and safety outcomes. These changes arise not from individual traits but from communication design embedded in routines, which is why OCC serves as the proximal lens for team functioning in healthcare [14, 15].

This positioning aligns the present study with a mechanism based view. Loneliness is a relational strain that can depress trust and voice, two pillars of OCC. Testing OCC as the outcome, therefore, targets a unit-level lever that managers can assess and improve through communication routines, while allowing hypothesis tests that connect individual relational experience to collective communication conditions.

**Mechanisms linking loneliness to OCC, why ED and LSC matter**

Emotional deprivation, ED, captures a deficit in close and trusting bonds at work. This deficit weakens the core conditions of the organizational communication climate, OCC. Lower trust reduces the willingness to share emerging problems. Reduced psychological safety suppresses speaking up and error reporting. Information becomes slower and less accurate. These are defining elements of OCC in clinical teams that depend on timely coordination and clear handoffs. Reviews and unit level studies in healthcare show that strained relationships and poor voice are associated with weaker communication climates and safety shortfalls [11, 16, 17]. Therefore, ED should predict a colder OCC because relational strain narrows the bandwidth for open, accurate, and safe communication during everyday patient work. Therefore, Hypothesis 1 was developed:

**H1.** Loneliness at work due to emotional deprivation is negatively associated with organizational communication climate.

Lack of social companionship, LSC, captures the scarcity of day-to-day affiliative contact at work. This facet matters for OCC because informal interaction is a carrier of weak ties, quick clarifications, and coordination cues across roles and shifts. When LSC is high, informal channels thin and transactive memory becomes fragmented. Teams lose opportunities to check understanding in the flow of work. Evidence from recent healthcare and organizational samples links loneliness to modifiable features of work design and interaction routines, which positions OCC as a proximal outcome lens for companionship deficits in unit based care delivery [1, 6, 10]. In practice, thinner informal channels are visible as delayed escalation, guarded talk, and more handoff frictions, all signals of a weaker OCC. Therefore, Hypothesis 2 was developed:

**H2.** Loneliness at work due to lack of social companionship is negatively associated with organizational communication climate.

**Perceived social competence as a boundary condition**

Perceived social competence reflects individuals’ beliefs that they can initiate, sustain, and repair interpersonal exchanges, and that they can communicate effectively when demands are high. Social Cognitive Theory proposes that people integrate what they observe in social settings with their self-perceptions and efficacy beliefs, which then guide how they adapt to situational demands and regulate behavior in relationships [18, 19]. In teams that operate under time pressure, stronger competence beliefs should help employees maintain clarity, seek help early, and frame problems as manageable, which supports the openness and accuracy that define the organizational communication climate.

Evidence aligns with this mechanism. Employees with higher social competence report more effective workplace communication and better adjustment under strain, which translates into collaborative behaviors that sustain a positive climate [20, 21]. Studies that track competence alongside work stressors show that communication competence predicts beneficial employee outcomes and buffers the effects of relational strain on performance and well-being, consistent with a stress-buffering role in social exchanges [2, 22]. In healthcare units, where rapid coordination and psychological safety are essential, competence beliefs should preserve trust, information sharing, and voice even when employees feel relationally deprived.

Applied to the present model, perceived social competence functions as a personal resource that can interrupt the pathway from emotional deprivation to a colder communication climate. When competence is high, employees are more likely to articulate needs, invite feedback, and join coordination routines, which offsets the tendency of emotional deprivation to reduce speaking up and narrow information flow. When competence is low, relational deficits are more likely to translate into guarded talk and slower escalation, which weakens the climate. Therefore, Hypothesis 3 was developed:

**H3.** Perceived social competence moderates the association between emotional deprivation and organizational communication climate, such that the negative association is weaker at higher levels of perceived social competence.

Lack of social companionship reflects scarce day-to-day affiliative contact at work. Employees miss casual conversation during breaks, quick check ins at handoffs, and routine small talk that maintains weak ties. This scarcity fuels isolation and reduces initiative in everyday communication. In clinical units, thin informal channels mean fewer timely clarifications and fewer spontaneous coordination cues, which lowers openness and slows information flow in the organizational communication climate. Recent healthcare studies tie speaking up and communication conditions to patient safety outcomes, which supports treating companionship scarcity as a pathway to climate degradation in unit-based care delivery [23, 24].

Perceived social competence can counter this pathway. Employees who believe they can initiate and sustain interactions are more likely to start brief check ins, ask for micro feedback, and join recurring communication routines such as safety huddles and short debriefs. These behaviors rebuild weak ties and keep information circulating despite limited companionship opportunities. Empirical work in health services shows strong links between effective communication and teamwork, and broad reviews connect quality communication to better patient centered outcomes, which is consistent with a buffering effect on relational strain [25, 26]. Therefore, Hypothesis 4 was developed:

**H4.** Perceived social competence moderates the association between lack of social companionship and organizational communication climate.

**Position of demographic characteristics**

Gender, marital status, education, profession, and clinical unit shape interaction opportunities and support in hospitals. Evidence shows patterned differences that matter for how staff perceive and enact communication. For gender and marital status, a UK study during COVID-19 reported that “married women had lower levels of self-reported wellbeing than married men” and that “married staff overall perceived their wellbeing as significantly higher than single members of staff” [27].

Profession and unit context also matter. A recent meta-analysis noted that failures in interprofessional collaboration “were the primary cause of adverse medical events” and highlighted contextual barriers such as “a stressful hospital environment” and “a lack of defined roles” [28].

Clinical units differ in tempo and coordination demands. A qualitative study of emergency and intensive care nurses described shared pressures that require “rapid decisions under pressure” and strong teamwork patterns, with managerial emphasis on creating “a supportive work environment” to sustain communication quality [29].

These demographic differences can confound focal relationships between loneliness and the organizational communication climate. To isolate mechanisms, demographics are included as covariates rather than core moderators. This keeps the model centered on how emotional deprivation and lack of social companionship map onto the communication climate, and how perceived social competence buffers these links. Where useful, exploratory interaction checks will be run and documented outside the core model. This approach aligns with evidence that unit stressors, role structures, and collaboration barriers vary by context and can shift climate perceptions without representing the theorized mechanism itself [28]. Accordingly, Hypothesis 5 and 6 are framed as exploratory probes rather than core theory tests and are reported outside the main results if estimated.

**H5.** Demographic characteristics (gender, marital status, educational attainment, profession, and work unit), in conjunction with perceived social competence, may moderate the association between emotional deprivation and organizational communication climate.

**H6.** Demographic characteristics (gender, marital status, educational attainment, profession, and work unit), in conjunction with perceived social competence, may moderate the association between lack of social companionship and organizational communication climate.

**References**

1. Bryan BT, Andrews G, Thompson KN, Qualter P, Matthews T, Arseneault L. Loneliness in the workplace: a mixed-method systematic review and meta-analysis. Occup Med (Chic Ill). 2023;73:557–67.

2. McCarthy JM, Erdogan B, Bauer TN, Kudret S, Campion E. All the Lonely People: An Integrated Review and Research Agenda on Work and Loneliness. J Manage. 2025;0:01492063241313320.

3. Sarason IG. Social support: Theory, research and applications. Springer Science & Business Media; 2013.

4. Gerson AC, Perlman D. Loneliness and expressive communication. Journal of Abnormal Psychology. 1979;88:258–61.

5. Segrin C. Interpersonal communication problems associated with depression and loneliness. In: Andersen PA, Guerrero LKBT-H of C and E, editors. San Diego: Academic Press; 1996. p. 215–42.

6. Jung Y-S, Jung H-S, Yoon H-H. The Effects of workplace loneliness on the psychological detachment and emotional exhaustion of hotel employees. International Journal of Environmental Research and Public Health. 2022;19.

7. Ozcelik H, Barsade SG. No employee an island: Workplace loneliness and job performance. Acad Manag J. 2018;61:2343–66.

8. Wright S. The future of work loneliness research. Curr Opin Behav Sci. 2025;65:101571.

9. Hower KI, Vennedey V, Hillen HA, Stock S, Kuntz L, Pfaff H, et al. Is Organizational Communication Climate a Precondition for Patient-Centered Care? Insights from a Key Informant Survey of Various Health and Social Care Organizations. International Journal of Environmental Research and Public Health. 2020;17.

10. Lee C-C, Zheng Y-R, Yeh W-C, Yu Z. The influence of communication climate, organizational identification, and burnout on real estate agents’ turnover intention. Humanit Soc Sci Commun. 2023;10:641.

11. Wieke Noviyanti L, Ahsan A, Sudartya TS. Exploring the relationship between nurses’ communication satisfaction and patient safety culture. J Public health Res. 2021;10.

12. van Zoonen W, Sivunen AE, Blomqvist K. Out of sight – Out of trust? An analysis of the mediating role of communication frequency and quality in the relationship between workplace isolation and trust. Eur Manag J. 2024;42:515–26.

13. Domènech-Abella J, Mundó J, Haro JM, Muntaner C. Workplace and non-workplace loneliness: a cross-sectional comparative study on risk factors and impacts on absenteeism and mental health among employees in Spain. Soc Psychiatry Psychiatr Epidemiol. 2025. https://doi.org/10.1007/s00127-025-02899-z.

14. National Health Service [NHS] England. Improving Patient Safety Culture A practical guide In association with. 2023.

15. American Hospital Association [AHA]. Insights Report: Improvement in Safety Culture Linked to Better Patient and Staff Outcomes. 2025.

16. Wright S, Silard A. Unravelling the antecedents of loneliness in the workplace. Hum Relations. 2020;74:1060–81.

17. Keshtkar L, Bennett-Weston A, Khan AS, Mohan S, Jones M, Nockels K, et al. Impacts of Communication Type and Quality on Patient Safety Incidents. Ann Intern Med. 2025;178:687–700.

18. Bandura A. Social foundations of thought and action: A social cognitive theory. Englewood Cliffs , NJ; 1986.

19. Luszczynska A, Schwarzer R. Social cognitive theory. Fac Heal Sci Publ. 2015;2015:225–51.

20. Tripathy M. Relevance of soft skills in career success. MIER J Educ Stud Trends Pract. 2020;10 1 SE-Articles:91–102.

21. Nwanzu CL, Babalola SS. The Effect of work-life balance and social competence on the psychological well-being of public service employees. Int J Manag Entrep Soc Sci Humanit. 2023;6 2 SE-Research Articles:56–70.

22. Baron RA, Markman GD. Beyond social capital: the role of entrepreneurs’ social competence in their financial success. J Bus Ventur. 2003;18:41–60.

23. Soyer Er Ö, Gül İ. The Speaking Up Climate of Nurses for Patient Safety Concerns and Unprofessional Behaviors: The Effects of Teamwork and Safety Climate. J PeriAnesthesia Nurs. 2024;39:782–8.

24. Lai Y-H, Chang C-W, Wu M-J, Chen H-H, Lin S-P, Chin C-S, et al. Effectiveness of Huddles in Improving the Patient Safety Attitudes Among Clinical Team Members. Qual Manag Health Care. 2024;33:239–45.

25. Meneses-La-Riva ME, Fernández-Bedoya VH, Suyo-Vega JA, Ocupa-Cabrera HG, Grijalva-Salazar RV, Ocupa-Meneses G di D. Enhancing Healthcare Efficiency: The Relationship Between Effective Communication and Teamwork Among Nurses in Peru. Nurs Reports. 2025;15:1–13.

26. Sharkiya SH. Quality communication can improve patient-centred health outcomes among older patients: a rapid review. BMC Health Serv Res. 2023;23:1–14.

27. Peng J, Wu WH, Doolan G, Choudhury N, Mehta P, Khatun A, et al. Marital Status and Gender Differences as Key Determinants of COVID-19 Impact on Wellbeing, Job Satisfaction and Resilience in Health Care Workers and Staff Working in Academia in the UK During the First Wave of the Pandemic. Front Public Heal. 2022;10:1–11.

28. Moloro AH, Sabo KG, Lahole BK, Wengoro BF, Mare KU. Prevalence of interprofessional collaboration towards patient care and associated factors among nurses and physician in Ethiopia, 2024: a systematic review and meta-analysis. BMC Nurs. 2025;24:1–15.

29. Liu Y, Zheng D, Xiao Y, Li Y, Huang S, Xiong J. A qualitative study of emergency and intensive care unit nurses’ experience of workflow: I enjoy the “flow” at work. BMC Nurs. 2025;24:1–10.
