# Supplementary material for: Workplace loneliness and the communication climate of healthcare workers: the moderating role of perceived social competence
Source: BMC Health Serv Res. 2025 Dec 18;26:106. doi: 10.1186/s12913-025-13911-2 (PMC12829023; doi:10.1186/s12913-025-13911-2)
Supplement: Supplementary file 2 — Supplementary Material 2 [file 12913_2025_13911_MOESM2_ESM.docx]

**SUPPLEMENTARY FILE-S2**

**Construct validity**

Confirmatory factor analysis (CFA) is an important method used to assess the construct validity of measurement instruments and the fit of theoretical models to data (Byrne, 2013). In this study, CFA was conducted for the Loneliness at Work, Organizational Communication Climate, and Perceived Social Competence scales using the IBM AMOS 23 program. According to the results of the analysis, the model fit indices of the scales used were generally acceptable (Table S2.1). The x²/df, GFI, AGFI, CFI, NFI, TLI, SRMR and RMSEA values, which are among the fit criteria, were found to be within the recommended limits, which supported the construct validity of the scales [3, 4].

**Table S2.1.** Confirmatory factor analysis fit indices for all scales, x²/df, GFI, AGFI, CFI, NFI, TLI, SRMR, RMSEA, and recommended thresholds.

| **Scales** | **x^2/^df** | **GFI** | **AGFI** | **CFI** | **NFI** | **TLI** | **SRMR** | **RMSEA** |
| --- | --- | --- | --- | --- | --- | --- | --- | --- |
| Loneliness in Work Life | 3.059 | 0.913 | 0.882 | 0.971 | 0.957 | 0.965 | 0.038 | 0.073 |
| Organizational Communication Climate | 2.446 | 0.855 | 0.832 | 0.973 | 0.955 | 0.971 | 0.011 | 0.061 |
| Perceived Social Competence | 1.468 | 0.992 | 0.975 | 0.999 | 0.998 | 0.998 | 0.003 | 0.035 |
| Recommended values | ≤ 5 | ≥ 0.8 | ≥ 0.8 | ≥ 0.9 | ≥ 0.9 | ≥ 0.9 | ≤ 0.9 | ≤ 0.08 |

**Notes:** x^2^/df: Chi-square to degree of freedom; GFI: Goodness of fit index; NFI: Normative fit index; TLI: Tucker–Lewis’s index; AGFI: Adjusted goodness of fit index; CFI: Comparative fit index; RMSEA: Root mean square error of approximation; and SRMR: Standardized root mean residual

In particular, the Perceived Social Competence Scale showed strong construct validity with the best performance in all fit indices. However, the GFI and AGFI values for the Organizational Communication Climate Scale were found to be borderline acceptable. The results indicated that the scales used generally provided valid and reliable model fit [5]. These findings are consistent with the recommendations in the literature and indicate that the scales used work in an integrated manner with the theoretical models.

**References**

1. Byrne BM. Structural equation modeling with Mplus: Basic concepts, applications, and programming. routledge; 2013.

2. Hair JF, Black WC, Babin BJ, Anderson RE. Multivariate data analysis: A global perspective. 2010.

3. Hu L, Bentler PM. Cutoff criteria for fit indexes in covariance structure analysis: Conventional criteria versus new alternatives. Struct Equ Model A Multidiscip J. 1999;6:1–55.

4. Awang P. SEM made simple: A gentle approach to learning Structural Equation Modeling. MPWS Rich Publication, Bangi; 2015.

5. Bentler PM, Bonett DG. Significance tests and goodness of fit in the analysis of covariance structures. Psychol Bull. 1980;88:588.
